# Performance of PRECISE-DAPT and Age–Bleeding–Organ Dysfunction Score for Predicting Bleeding Complication During Dual Antiplatelet Therapy in Chinese Elderly Patients

**DOI:** 10.3389/fcvm.2022.910805

**Published:** 2022-07-08

**Authors:** Liang Dong, Cao Lu, Chen Wensen, Chen Fuzhong, Muhammad Khalid, Dong Xiaoyu, Li Guangjuan, Qian Yanxia, Zhang Yufeng, Liu Xinjian, Chen Leilei, Wang Junhong

**Affiliations:** ^1^Department of Cardiology, The First Affiliated Hospital of Nanjing Medical University, Nanjing, China; ^2^Office of Infection Management, The First Affiliated Hospital of Nanjing Medical University, Nanjing, China; ^3^Department of Cardiology, Xinjiang Yili Friendship Hospital, Yili Kazak Autonomous Prefecture, Xinjiang, China

**Keywords:** bleeding risk scores, percutaneous coronary intervention, dual-antiplatelet therapy, PRECISE-DAPT, ABO score

## Abstract

**Background:**

Recently, the Age–Bleeding–Organ Dysfunction (ABO) algorithm was recommended by the Asian Pacific Society of Cardiology Consensus as a binary approach to evaluate bleeding risk. This analysis made comparison of the predictive performances between the PRECISE-DAPT and ABO bleeding score in identifying the risk of 12-months major bleeding in Chinese elderly patients over 65 years old patients who underwent percutaneous coronary intervention (PCI) during dual-antiplatelet therapy period.

**Methods:**

A total of 2,037 elderly coronary artery disease (CAD) patients (≥65 years) receiving dual antiplatelet therapy (DAPT) after PCI were enrolled in the study. The predictive accuracy of the two bleeding risk scores (PRECISE-DAPT and ABO) was compared for identifying the risk of bleeding during the dual-antiplatelet therapy in patients who underwent PCI. Major clinically relevant bleeding events were defined according to the Bleeding Academic Research Consortium (BARC) criteria.

**Results:**

The PRECISE-DAPT score in the no bleeding, BARC = 1 bleeding, BARC ≥ 2 bleeding patients was 23.55 ± 10.46, 23.23 ± 10.03, and 33.54 ± 14.33 (*p* < 0.001), respectively. Meanwhile, the ABO score in the three groups was 0.72 ± 0.80, 0.69 ± 0.81, and 1.49 ± 0.99 (*p* < 0.001), respectively. The C-statistic of the PRECISE-DAPT model for prediction of BARC ≥ 2 bleeding in overall patients was 0.717 (95% CI, 0.656–0.777) and 0.720 (95% CI, 0.656–0.784) in acute coronary syndrome (ACS) patients. Similar discriminatory capacity was demonstrated in the ABO risk score [overall, patients, AUC: 0.712 (95% CI, 0.650–0.774); ACS patients, AUC: 0.703 (95% CI, 0.634–0.772)]. No differences were observed when the ABO model was in comparison with the PRECISE-DAPT model, regardless in overall patients (*z* = −0.199, *p* = 0.842) or ACS patients (*z* = −0.605, *p* = 0.545). The calibration for BARC ≥ 2 bleeding of the PRECISE-DAPT and ABO score were acceptable, regardless in overall patients [goodness-of-fit (GOF) Chi-square = 0.432 and 0.001, respectively; *p*-value = 0.806 and 0.999, respectively] or ACS patients (GOF Chi-square = 0.008 and 0.580, respectively; *p*-value = 0.996 and 0.748, respectively).

**Conclusion:**

No matter of clinical presentation in Asian 65-years older patients with DAPT, the PRECISE-DAPT, and ABO scores had the similar discriminative ability for 12-months BARC ≥ 2 bleeding. Considering the simplicity and reliability, the PRECISE-DAPT score might be more clinically applicable in the overall population and ACS patients in bleeding prediction.

## Introduction

Cardiovascular diseases (CVDs), consisting of acute (ACS) or chronic coronary syndromes (CCS) and a diverse range of other CVDs, pose the leading cause of worldwide mortality and significantly contribute to reduce quality of life (1, 2). While the combination of anti-thrombotic medications such as aspirin and P2Y12 inhibitors, and the use of invasive risk stratification such as percutaneous coronary intervention (PCI) at early stage has improved the outcomes of ACS and CCS patients, the postoperative bleeding risk, especially during the dual-antiplatelet therapy period, remains a significant adverse complication (3). The bleeding complication is a common serious complication after PCI and during the duration of dual antiplatelet therapy (DAPT), notably in elderly patients, because the elderly frequently displays high possibility of more vessel’s lesions and life-threatening bleeding and other complex comorbidities (4, 5). Furthermore, it is also combined with incremental morbidity and mortality (6).

Up to now, various bleeding risk models have been confirmed to predict bleeding events at early and late stage, such as the CRUSADE (7, 8) and ACUITY (9, 10), PRECISE-DAPT, and ARC–HBR (11, 12) bleeding risk scores. Due to the unique and distinct characteristics of ACS/CCS patients in the Asia-Pacific region, including differences in clinical characteristics, the invasiveness and complexity of the procedure and low number of observed patients from the Asia-Pacific region in pivotal research on bleeding, the above mentioned bleeding risk scores, therefore, may not be directly applicable to these populations (13). The Age–Bleeding–Organ Dysfunction (ABO) was recently proposed as a binary approach to assist clinicians to evaluate bleeding risk of Asian Pacific Society and guide treatment by The Asian Pacific Society of Cardiology (14).

In order to guide clinical practice and, if possible, improve our awareness about bleeding complications, our study tried to evaluate and compare the performances of the PRECISE-DAPT, and ABO scores for predicting 12 months serious bleeding in coronary artery disease (CAD) patients receiving DAPT after PCI therapy.

## Materials and Methods

### Study Population

This was a multi-centered, retrospective study conducted among the patients who were diagnosed as coronary heart disease and received DAPT after PCI from the first affiliated hospital with Nanjing Medical University, the first affiliated Hospital of Soochow University, and the affiliated hospital of Yangzhou University from September 2016 to July 2018. All patients who met the inclusion criteria were retrospectively selected from the electronic medical records database at above centers. Inclusion criteria were as follows: (1) age equal to or older than 65 years, (2) all the patients received 1-year DAPT therapy, except for some of them changed to single antiplatelet therapy (SAPT) treatment due to bleeding complications. Patients were excluded for the following reasons: (1) patients that had contraindications to DAPT therapy such as active bleeding, peptic ulcer or were allergic to one or more antiplatelet drugs; (2) patients with hematological disorders; (3) patients with anticoagulation indications receiving oral anticoagulation therapy within 1 year after PCI according to the current principles of treatment; (4) the patients were complicated with serious diseases such as malignant tumor with less than 1 year life expectancy; and (5) incomplete follow-up data due to loss of contact. Furthermore, patients with a history of PCI intervention or coronary artery bypass surgery within 1 year before enrollment were also excluded in order to rule out possible increased bleeding risks associated with prolonged DAPT. Finally, there were 2,037 patients constituted the study population.

The details of clinical characteristics, antithrombotic therapy, angiography parameters, biochemical, blood routine test, and electrocardiography were collected retrospectively from the hospital medical electronic database at the time of index PCI (15). The study protocol was conducted in accordance with the Declaration of Helsinki and was approved by the institutional ethics committee of each participating center (No. 2020-SR-472). The informed consent of the participants was waived in the study and approved by our ethical committee.

### Variable Definition

At the time of enrollment into the study, the patients’ clinical data were recorded, and the PRECISE-DAPT (16) and ABO (14) scores were calculated. The PRECISE-DAPT score was composed of five variables, such as age, hemoglobin, white blood cell count, creatinine clearance, and previous spontaneous bleeding. The ABO score was constituted by three category variables, including age (Frail elderly >75 years, advanced age >85 years, and life expectancy <1 year), bleeding (spontaneous intracranial hemorrhage, recurrent gastrointestinal bleeding, and hemoglobin <9 g/dl), organ dysfunction (liver cirrhosis, end-stage renal failure requiring dialysis, and bone marrow failure, e.g., severe thrombocytopenia, platelet count <50,000/μl, and stroke in the last 6 months) (14). Because the ABO score is a qualitative variable of binary classification, in order to facilitate comparison with the PRECISE-DAPT scores, we define each risk factor as 1 point, and then add each point for comparison. The total bleeding risk scores of PRECISE-DAPT were assessed using an online calculator by entering all variables for each patient.^1^ The predictive accuracy of the two bleeding risk scores was compared to identifying the bleeding risk during the dual-antiplatelet therapy in ACS/CCS patients undergoing PCI.

### Endpoint Definition

The enrolled patients were followed up by a clinical visit supported by the phone interviewer (at least 1 year) after admission for the PCI in order to obtain the endpoint information. According to the Bleeding Academic Research Consortium (BARC) criteria, the clinically relevant bleeding events were defined as type 2 to 5 to compare the predictive accuracy of these scores (17). In addition, the patients with BARC 3 and 5 type bleeding events were further analyzed due to its serious clinical consequence. Details of the BARC criteria have been reported elsewhere (18). The information of bleeding sites, the levels of admission hemoglobin and nadir hemoglobin, and transfusion of red blood cells were recorded in our study, and clinically relevant bleeding events were defined as BARC 2 to BARC 5 bleeding (excluding BARC 4), no bleeding was defined as BARC 0 and minor and unactionable bleeding that does not need patients to seek unscheduled professional treatment was defined as BARC 1.

### Statistical Analysis

The patients were classified in three risk categories for bleeding (no bleeding, BARC < 2 type bleeding and BARC ≥ 2 type bleeding and their characteristics were reported by descriptive statistics. Quantitative variables are reported as standard deviation (mean ± SD) or interquartile range, and qualitative variables are expressed as absolute numeric values and percentages. The χ^2^ test or Fisher Exact test was appropriately compared between quantitative and qualitative variables, and separate analysis was conducted among the patients’ diagnosed as CCS and ACS. A statistically significant difference indication was considered as a two-sided *p*-value < 0.05. Receiver operating characteristics (ROC) curves were used to evaluate the discriminative capacities of the two scores, and C-statistics greater than 0.7 was considered as an acceptable discriminatory capacity (19). The calibration of the models was evaluated using the Hosmer–Lemeshow goodness-of-fit (GOF) statistical test (20). A significant *p*-value less than 0.05 indicated a poor calibration. The C-statistics for the two risk models were compared to each other using the DeLong test (21). All data were processed by SPSS (version 26.0) statistical software and R language software (version 3.6.0).

## Results

### Baseline Characteristics

**FIGURE 1:**
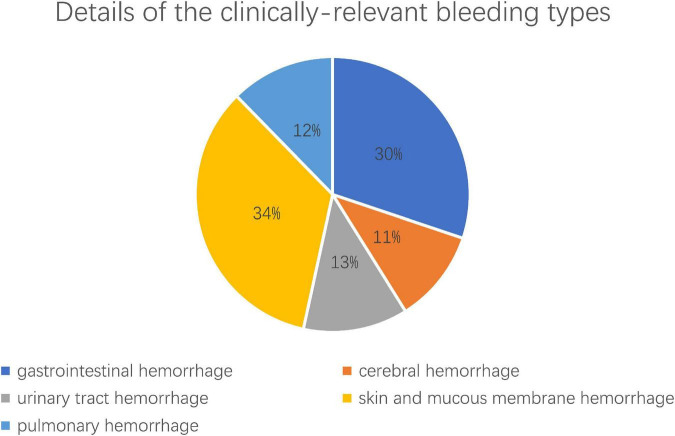
The details of the clinically relevant bleeding types after PCI. Clinically relevant bleeding complications (BARC = type 2–5, excluding BARC 4) including 22 (30%) cases of gastrointestinal hemorrhage, 8 (11%) cases of cerebral hemorrhage, 9 (13%) cases of urinary tract hemorrhage, 25 (34%) cases of skin and mucous membrane hemorrhage, and 9 (12%) cases of pulmonary hemorrhage.

Table 1 shows the baseline characteristics of the study. The overall population age was 73.76 ± 6.00 years old, and women was 29.81%. Patients presented by ACS in 84.63% of cases, CCS in 15.37% of cases. Bleeding complications occurred in 202 patients, corresponding to an overall bleeding rate of almost 10%, BARC ≥ 2 bleeding complications occurred in 73 patients, among which, 22 cases of gastrointestinal hemorrhage, 8 cases of cerebral hemorrhage, 9 cases of urinary tract hemorrhage, 25 cases of skin and mucous membrane hemorrhage, and 9 cases of pulmonary hemorrhage. In addition, one type 5 and 10 BARC type 3 patients were recorded in our enrolled patients. Figure 1 shows details of the clinically relevant bleeding types. According to the above BARC bleeding criteria, the patients were stratified in three risk categories for bleeding: no bleeding group (*n* = 1,835), BARC < type 2 group (*n* = 129) and BARC ≥ type 2 group (*n* = 73). Among the three groups, age, history of myocardial infarction, cerebral infarction, cerebral hemorrhage, peptic ulcer bleeding and heart failure, the level of triglyceride, diastolic blood pressure, and creatinine, the number of diseased vessels, the score of PRECISE-DAPT score and ABO, the usage of PPI were statistically different (*p* < 0.05).

**TABLE 1:** Baseline clinical characteristics and in-hospital management of study population.

	No bleeding	BARC < 2	BARC ≥ 2	*P*-value
	*n* = 1,835	*n* = 129	*n* = 73	
**Demographic data**				
Age, years	73.76 ± 6.00	73.61 ± 6.41	78.17 ± 5.99	< 0.001
Male (%)	1,288 (70.19)	84 (65.12)	50 (68.49)	0.437
**Previous history**				
Hypertension (%)	1,329 (72.43)	97 (75.20)	56 (76.71)	0.513
Diabetes mellitus (%)	477 (25.99)	34 (26.36)	25 (34.25)	0.238
Smoking history (%)	685 (37.33)	51 (39.53)	24 (32.88)	0.605
Family history of CHD (%)	40 (2.18)	3 (2.33)	1 (1.37)	0.886
Previous MI (%)	109 (5.94)	10 (7.75)	10 (13.70)	0.021
Previous PCI (%)	247 (13.46)	12 (9.30)	13 (17.81)	0.236
Previous CABG (%)	21 (1.14)	2 (1.55)	1 (1.37)	0.901
Previous ischemic stroke (%)	169 (9.21)	15 (11.63)	15 (20.55)	0.004
Encephalorrhagia (%)	8 (0.44)	4 (3.10)	6 (8.22)	< 0.001
Peptic ulcer bleeding (%)	50 (2.78)	2 (1.50)	8 (10.96)	< 0.001
peripheral artery disease (%)	20 (1.09)	1 (0.78)	0 (0)	0.641
Chronic renal insufficiency (%)	51 (2.78)	3 (2.32)	3 (4.11)	0.675
Heart failure (%)	34 (1.88)	2 (1.55)	5 (6.85)	0.01
Atrial fibrillation (%)	77 (4.20)	6 (4.65)	3 (4.11)	0.937
**Admission data**				
SBP, mmHg	133.61 ± 20.43	134.62 ± 20.16	136.47 ± 21.25	0.451
DBP, mmHg	77.23 ± 11.87	75.02 ± 10.74	75.06 ± 12.67	0.045
Heart rate, bpm	74.42 ± 13.26	74.19 ± 11.71	73.88 ± 11.56	0.927
Hgb, g/L	131.06 ± 17.95	131.01 ± 16.85	126.93 ± 15.28	0.155
PLT, ×10^9^/L	187.68 ± 62.82	198.31 ± 70.30	198.42 ± 63.32	0.077
WBC, ×10^9^/L	7.30 ± 3.22	7.59 ± 3.29	7.62 ± 2.59	0.447
TC, (mmol/L)	4.07 ± 1.11	4.04 ± 1.07	4.03 ± 1.07	0.920
TG, (mmol/L)	1.54 ± 0.93	1.41 ± 0.78	1.23 ± 0.47	0.008
LDL-C, (mmol/L)	2.38 ± 0.89	2.37 ± 0.87	2.41 ± 0.91	0.969
HDL-C, (mmol/L)	1.11 ± 0.34	1.10 ± 0.28	1.08 ± 0.30	0.654
Ccr, ml/min/1.73m^2^	67.31 ± 22.29	70.77 ± 21.48	55.82 ± 24.01	< 0.001
PT, s	12.80 ± 1.86	12.73 ± 1.36	13.01 ± 1.59	0.567
**Diseased vessels**				0.016
Single vessel (%)	1,366 (74.44)	88 (68.22)	49 (67.12)	
Two vessels (%)	410 (22.34)	30 (23.26)	18 (24.66)	
Three vessels (%)	59 (3.22)	11 (8.53)	6 (8.22)	
Coronary stent number, *n*	1.58 ± 0.90	1.74 ± 1.11	1.69 ± 0.93	0.085
PRECISE-DAPT score	23.55 ± 10.46	23.23 ± 10.03	33.54 ± 14.43	< 0.001
ABO score	0.72 ± 0.80	0.69 ± 0.81	1.49 ± 0.99	< 0.001
**Antithrombotic treatment**				0.326
Aspirin + clopidogrel, (%)	1,308 (71.28)	91 (70.54)	58 (79.45)	
Aspirin + ticagrelor, (%)	527 (28.77)	38 (29.46)	15 (20.55)	
**Other medications**				
RAS inhibitors, (%)	712 (38.80)	53 (41.09)	32 (43.84)	0.588
B -blockers, (%)	994 (54.17)	82 (63.57)	37 (50.68)	0.058
Proton-pump inhibitor, (%)	756 (41.20)	53 (41.09)	43 (58.90)	0.007

*Data are expressed as mean ± SD, medians (25th–75th percentiles), or number (percentage). BARC, Bleeding Academic Research Consortium; MI, myocardial infarction; PCI, percutaneous coronary intervention; CABG, coronary artery bypass grafting; NSTEMI, non–ST-segment elevation myocardial infarction; STEMI, ST-segment elevation myocardial infarction; SBP, systolic blood pressure; DBP, diastolic blood pressure; HR, heart rate; Hgb, hemoglobin; PLT, platelets; WBC, white blood count; TC, total cholesterol; TG, triglyceride; LDL-C, low-density lipoprotein cholesterol; HDL, high-density lipoprotein cholesterol; cCr, creatinine clearance rate; PT, prothrombin time; ACEI, angiotensin converting enzyme inhibitor; ARB, angiotensin receptor blocker; TT, triple therapy; PPI, proton pump inhibitor.*

In comparison with no bleeding group, patients of BARC ≥ type 2 group had a lower creatinine clearance rate (55.82 ± 24.01 vs. 67.31 ± 22.29 ml/min/1.73 m^2^, *p* < 0.001), lower level of triglyceride (1.22 ± 0.47 vs. 1.54 ± 0.93 mmol/L, *p* = 0.006), more advanced age (78.17 ± 5.99 vs. 73.76 ± 6.00, *p* < 0.001), higher ABO score (1.49 ± 0.99 vs. 0.72 ± 0.80, *p* < 0.001), and PRECISE-DAPT score (33.54 ± 14.33 vs. 23.55 ± 10.46, *p* < 0.001). More patients with BARC ≥ 2 type bleeding events had a history of myocardial infarction (13.70 vs. 5.94%, *p* = 0.007), cerebral infarction (20.55 vs. 9.21%, *p* = 0.001), cerebral hemorrhage (8.22 vs. 0.44%, *p* < 0.001), peptic ulcer (10.96 vs. 2.78%, *p* < 0.001), heart failure (6.85 vs. 1.88%, *p* = 0.003) and PPI usage after PCI (58.90 vs. 41.20%, *p* = 0.002) than the patients without bleeding events. In addition, patients of BARC ≥ type 2 group have a higher ABO score (1.49 ± 0.99 vs. 0.69 ± 0.81, *p* < 0.001) and PRECISE-DAPT score (33.54 ± 14.33 vs. 23.23 ± 10.03, *p* < 0.001) than the patients in BARC < type 2 group.

### The Performance of Bleeding Prediction in the PRECISE-DAPT and Age–Bleeding–Organ Dysfunction Risk Score

In the study, we calculated the PRECISE-DAPT and ABO scores of 2,037 patients who underwent PCI. The PRECISE-DAPT score in the no bleeding, BARC < type 2 bleeding, BARC ≥ type 2 patients were 23.55 ± 10.46, 23.23 ± 10.03, and 33.54 ± 14.33 (*p* < 0.001), respectively. Meanwhile, the ABO score in the three groups was 0.72 ± 0.80, 0.69 ± 0.81, and 1.49 ± 0.99 (*p* < 0.001), respectively. The C-statistic of the PRECISE-DAPT model for prediction of BARC ≥ 2 bleeding in overall patients was 0.717 (95% CI, 0.656–0.777) and in ACS patients was 0.720 (95% CI, 0.656–0.784). The cutoff value of the PRECISE-DAPT model for prediction of BARC ≥ 2 bleeding in overall patients was 25 while in ACS patients was 32. In the overall population and ACS patients, respectively, the ABO risk score (C-statistics: 0.712 and 0.703, respectively; 95% CI: 0.650–0.774 and 0.634–0.772, respectively) was demonstrated similar discriminatory capacity. The cutoff value of the ABO model for prediction of BARC ≥ 2 bleeding was 1 both in overall and ACS patients (Table 2).

**TABLE 2:** Discrimination for the two risk scores of 12-months after PCI BARC ≥ 2 bleeding events in overall and STEMI patients.

Risk group	Risk score	H-L test		C-statistic	95% CI
		χ 2	*p*		
Overall	PRECISE-DAPT	0.432	0.806	0.717	0.656–0.777
(*n* = 2,037)	ABO	0.001	0.999	0.712	0.650–0.774
ACS	PRECISE-DAPT	0.008	0.996	0.720	0.656–0.784
(*n* = 1,723)	ABO	0.580	0.748	0.703	0.634–0.772

### Comparison of the Bleeding Prediction Ability in the PRECISE-DAPT and Age–Bleeding–Organ Dysfunction Risk Score

**FIGURE 2:**
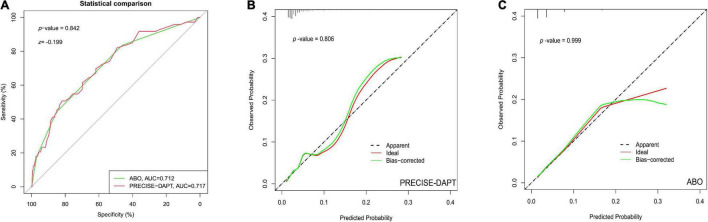
The performance of receiver operating characteristic (ROC) curve and calibration plot for PRECISE-DAPT and ABO risk score systems in overall patients with 1-year DAPT after PCI. **(A)** ROC curve for the prediction of BARC ≥ 2 type bleeding events by PRECISE-DAPT and ABO risk score systems in overall patients with 1-year DAPT after PCI [AUC: 0.717 and 0.712, respectively; (95% CI, 0.656–0.777)and (95% CI, 0.650–0.774), respectively; *p* < 0.001]. The C-statistics for the two risk models were compared to each other by the DeLong test (*z* = –0.199, *p* = 0.842). **(B)** The calibration plot for PRECISE-DAPT risk score in overall patients (GOF Chi-square = 0.432, *p*-value = 0.806). **(C)** The calibration plot for ABO risk score in overall patients (GOF Chi-square = 0.001 *p*-value = 0.999).

**FIGURE 3:**
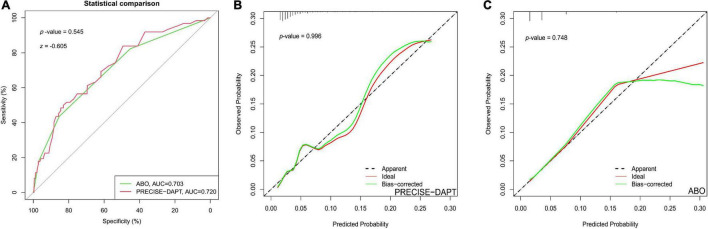
The performance of ROC curve and calibration plot for PRECISE-DAPT and ABO risk score systems in ACS patients with 1-year DAPT after PCI. **(A)** ROC curve for the prediction of BARC ≥ 2 type bleeding events by PRECISE-DAPT and ABO risk score systems in ACS patients with 1-year DAPT after PCI [AUC: 0.720 and 0.703, respectively; (95% CI, 0.656–0.784) and (95% CI, 0.634–0.772), respectively; *p* < 0.001]. The C-statistics for the two risk models were compared to each other by the DeLong test (*z* = –0.605, *p* = 0.545). **(B)** The calibration plot for the PRECISE-DAPT risk score in ACS patients (GOF Chi-square = 0.008, *p*-value = 0.996). **(C)** The calibration plot for the ABO risk score in ACS patients (GOF Chi-square = 0.580 *p*-value = 0.748).

**FIGURE 4:**
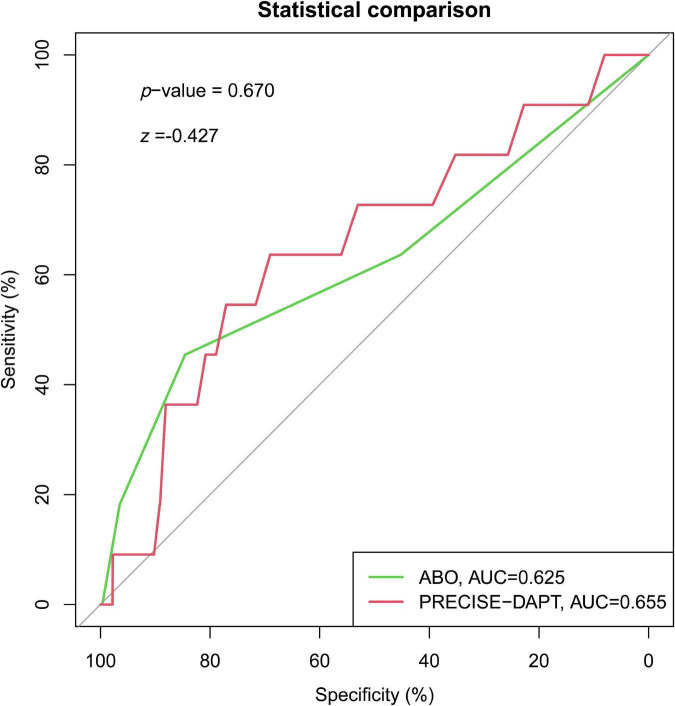
Receiver operating characteristic curve for the prediction of BARC = 3 or 5 type bleeding events by PRECISE-DAPT and ABO risk score systems in overall patients with 1-year DAPT after PCI [AUC: 0.655 and 0.625, respectively; (95% CI, 0.486–0.823) and (95% CI, 0.428–0.823), respectively; *p* = 0.076 and *p* = 0.152, respectively). The C-statistics for the two risk models were compared to each other by the DeLong test (*z* = –0.427, *p* = 0.670).

In order to compare the discriminative power of the two risk scores for predicting BARC ≥ 2 bleeding, the DeLong test was used to evaluate C-statistics each other. Table 3 showed that there were no statistical differences between the PRECISE-DAPT model and the ABO model in prediction of the BARC ≥ 2 bleeding events after PCI, regardless in overall patients (*z* = −0.199, *p* = 0.842) or ACS patients (*z* = −0.605, *p* = 0.545) (Figures 2 3). In addition, similar discriminative performance of the two risk score was observed in patients with BARC 3 and 5 bleeding events (Figure 4). The calibration abilities of the two risk scores were assessed by the Hosmer–Lemeshow GOF test. The calibration for BARC ≥ 2 bleeding of the PRECISE-DAPT and ABO score were acceptable, regardless in overall patients (GOF Chi-square = 0.432 and 0.001, respectively; *p*-value = 0.806 and 0.999, respectively) or ACS patients (GOF Chi-square = 0.008 and 0.580, respectively; *p*-value = 0.996 and 0.748, respectively) (Figures 2, 3).

**TABLE 3:** Comparisons of the discriminative power of the two risk scores for predicting BARC ≥ 2 bleeding.

Risk group	Comparison	*z*	*p*
Overall	PRECISE-DAPT vs. ABO	−0.199	0.842
(*n* = 2,037)			
ACS	PRECISE-DAPT vs. ABO	−0.605	0.545
(*n* = 1,723)			

## Discussion

Our study investigated the predictive performance between the PRECISE-DAPT and the ABO bleeding risk score, for 12-months clinically relevant bleeding complications post-PCI in Asian 65 year-older patients with DAPT. The main findings reported in this study can be summarized as follows: (1) No matter of clinical presentation, the PRECISE-DAPT, and ABO scores had adequate discriminative abilities (C-statistics: 0.70–0.75) for predicting 12-months BARC ≥ 2 bleeding after PCI. (2) Compared with the PRECISE-DAPT score, the ABO score showed a similar discriminative capacity for BARC ≥ 2 bleeding both in the overall and ACS patients.

Considering the higher mortality rate associated with bleeding manifestations, especially DAPT-related bleeding after PCI, bleeding prediction models play a more significant role in risk stratification and the choice of treatment. Approximately 11 years ago, the CRUSADE and ACUITY scores were designed to assess bleeding in ACS patients after PCI (8, 22). However, the bleeding predictive performance of the two risk scores should be updated, as the interventional procedure such as the access route of coronary angiography and anti-thrombotic medications have changed over time. Considering the changes, the PRECISE-DAPT score system was proposed in 2017 (6), which focused on the assessment of bleeding risk within 12 months after PCI. The short-term DAPT treatment was recommended for patients with score >25, which can outweigh the risks of extended treatment duration (23, 24), and we previously found that the PRECISE-DAPT score can predict BARC ≥ 2 bleeding events with a cutoff value of 32 in elderly patients over 75 (15). Consistently, this study found that the PRECISE-DAPT score remained efficient to predict 12-months BARC ≥ 2 bleeding in Asian elderly patients (≥65 years) receiving DAPT therapy. Even more, in the elderly ACS sub-group, the predictive performance of the PRECISE-DAPT score for clinically relevant bleeding was still efficient. Taken together, our results may indicate that PRECISE-DAPT is a feasible and reliable algorithm to evaluate the bleeding risk in Asian patients (aged ≥65 years) with DAPT within 12-month post-PCI. Furthermore, compared with patients on standard DAPT after drug-eluting stent implantation, the PRECISE-DAPT showed consistently prediction of bleeding risk, either in patients on ticagrelor monotherapy after 1-month DAPT or on aspirin monotherapy after 12-month DAPT (25). Even more, the 4-item PRECISE-DAPT score showed the similar discriminative ability to the 5-item iteration when white-blood-cell count was not available.

However, it was worth noting that, when compared with Western patients, the Asian patients had some special characteristics, such as lower systolic blood pressure (SBP) and heart rate, the amount of the prevalence of anemia and the rate of iatrogenic vessel access injury was relatively small (26–28). Therefore, the Age–Bleeding–Organ Dysfunction (“ABO”) algorithm was recently recommended by the Asian Pacific Society of Cardiology Consensus to evaluate bleeding risk in Asian stable CCS patients. In order to test the applicability of the new proposed recommendation in Asian elderly patients, we then analyzed the performance of the ABO algorithm in the prediction for the 1-year bleeding outcome in our study. Surprisingly, a similar discriminative performance of clinically relevant (BARC ≥ 2 type) or (BARC 3 or 5 type) bleeding during 12 months was observed in the ABO score when compared with the PRECISE-DAPT scores, irrespective in the overall population or ACS patients. Therefore, our results may indicate that the ABO score could be a candidate algorithm for Asian patients with clinically relevant bleeding prediction within 12-months DAPT post-PCI.

## Limitations

Several limitations in our study should be considered. Firstly, compared with other studies, this retrospective study had a relatively small sample size and a lower incidence of bleeding complications. Secondly, several drawbacks of the ABO algorithm should be mentioned before its clinical use. The ABO score lacks specific quantitative points and is relatively rough because of its binary approach, to facilitate calculating the bleeding risk of ABO score, and our study defines each risk factor as 1 point in the model. In fact, the weight of each risk factor is quite different (for example, both Frail elderly >75 years, advanced age >85 years, and life expectancy <1 year was 1 point when calculating the bleeding risk). Therefore, more samples are needed to calculate the weight of each risk factor in bleeding. Also, the ACS presentation *per se* has been confirmed to enhance major and actionable bleeding risks within 1 year after PCI (12, 29). The ABO score was originally designed to evaluate the bleeding risk in CCS patients and did not include the acute presentation, which may underestimate the risk of bleeding in ACS patients. Thirdly, the ABO algorithm, including those variables such as end-stage renal failure requiring dialysis or bone marrow failure (severe thrombocytopenia, platelet count <50,000/μl). Considering the high bleeding risk (HBR) after PCI in those kinds of patients, most of them will not receive PCI treatment or the intensity of antiplatelet therapy is usually relatively low in those patients. Therefore, there may be certain of selection bias when using the ABO algorithm to predict the bleeding after PCI in the real world. Consequently, considering the simplicity and reliability for calculation, the PRECISE-DAPT score might be more suitable than ABO score for clinical use in Asian patients at present. In addition, the recently developed ARC–HBR (the Academic Research Consortium for HBR) consortium was used to identify HBR patients after PCI, which has been validated the ability in large real-world cohorts of patients undergoing PCI for ACS and CCS (11, 29). Therefore, more consortiums, including ARC–HBR, PRECISE-DAPT, or ABO should be applied to identify the high bleeding patients after PCI in the future.

## Conclusion

No matter of clinical presentation, the PRECISE-DAPT, and ABO scores had possibly the similar discriminative ability for 12-months BARC ≥ 2 bleeding in Asian 65-year older patients with DAPT. Considering simplicity, reliability, and discriminative ability, the PRECISE-DAPT score might be more suitable in the overall population and ACS patients for the clinically relevant bleeding prediction of 12-months post-PCI. However, the ABO score also proves that along with the risk category, transition from low to high in 1 year, the probability of bleeding would increase subsequently.

This study would help clinicians make a distinction between different risk stratifications and provide assistance in the assessment and management of bleeding and ischemic risks in Asian ACS/CCS patients.

## Data Availability Statement

The raw data supporting the conclusions of this article will be made available by the authors, without undue reservation.

## Ethics Statement

The study protocol was conducted in accordance with Declaration of Helsinki and was approved by the Institutional Ethics Committee of each participating center (No. 2020-SR-472). Written informed consent for participation was not required for this study in accordance with the national legislation and the institutional requirements.

## Author Contributions

LD: conceptualization, methodology, software, investigation, formal analysis, and writing—original draft. CLu: data curation and writing—original draft. CW, CF, DX, and LG: visualization, investigation, data curation, and follow-up. MK: review and editing. QY and ZY: resources and supervision. LX, CLe, and WJ: visualization and writing—review and editing. WJ: conceptualization, funding acquisition, resources, supervision, and writing—review and editing. All authors contributed to the article and approved the submitted version.

## Conflict of Interest

The authors declare that the research was conducted in the absence of any commercial or financial relationships that could be construed as a potential conflict of interest.

## Publisher’s Note

All claims expressed in this article are solely those of the authors and do not necessarily represent those of their affiliated organizations, or those of the publisher, the editors and the reviewers. Any product that may be evaluated in this article, or claim that may be made by its manufacturer, is not guaranteed or endorsed by the publisher.

## Footnotes

1
http://www.precisedaptscore.com/predapt/
